# HIV-1 Vif and APOBEC3G: Multiple roads to one goal

**DOI:** 10.1186/1742-4690-1-28

**Published:** 2004-09-21

**Authors:** Joao Goncalves, Mariana Santa-Marta

**Affiliations:** 1 URIA-Centro de Patogénese Molecular, Faculdade de Farmácia, Universidade de Lisboa, 1649-019 Lisboa, Portugal

## Abstract

The viral infectivity factor, Vif, of human immunodeficiency virus type 1, HIV-1, has long been shown to promote viral replication *in vivo *and to serve a critical function for productive infection of non-permissive cells, like peripheral blood mononuclear cells (PBMC). Vif functions to counteract an anti-retroviral cellular factor in non-permissive cells named APOBEC3G. The current mechanism proposed for protection of the virus by HIV-1 Vif is to induce APOBEC3G degradation through a ubiquitination-dependent proteasomal pathway. However, a new study published in *Retrovirology *by Strebel and colleagues suggests that Vif-induced APOBEC3G destruction may not be required for Vif's virus-protective effect. Strebel and co-workers show that Vif and APOBEC3G can stably co-exist, and yet viruses produced under such conditions are fully infectious. This new result highlights the notion that depletion of APOBEC3G is not the sole protective mechanism of Vif and that additional mechanisms exerted by this protein can be envisioned which counteract APOBEC3G and enhance HIV infectivity.

## 

In contrast to most animal viruses, infection with the human and simian immunodeficiency viruses results in prolonged, continuous viral replication in the infected host. Remarkably, viral persistence is not thwarted by the presence of apparently vigorous, virus-specific immune responses. Several factors, including the evasion of an innate cellular anti-viral defense by HIV-1 as discussed in a recent *Retrovirology *article [1], are thought to contribute to persistent viral replication. Most notably during its course of engendering the development of acquired immunodeficiency syndrome (AIDS), HIV-1 mutates with high frequency and thus avoids immune response and intracellular defense mechanisms [2]. Interestingly, it has been observed for several years that the genomes of HIV-1, other retroviruses, and hepatitis B viruses show under certain conditions a very high rate of G-to-A hypermutation [2-5]. Earlier, this mutagenic phenomenon was attributed to the error-prone retroviral reverse transcriptase together with imbalances in the available deoxynucleotide pools in the cell. However, more recently a new player has been discovered, and new studies implicate the host cell cytidine deaminase APOBEC3G as responsible for G-to-A hypermutation in viral genomes [4,6].

APOBEC3G is a virion-encapsidated cellular protein that deaminates dC to dU in minus-strand viral cDNA during reverse transcription [7-10]. The uracil-containing cDNA may then activate a cellular uracil-DNA-glycosidase causing the failure of reverse transcription. This failure is characteristic of Vif-defective virus and results in the impairment of proviral integration into the host genome [10,11]. Furthermore, even if the reverse transcription is completed at low efficiency and the resulting proviral double stranded cDNA is integrated into the cellular genome, the massive C-U conversion in the minus strand leads to pervasive G to A hypermutation of the proviral plus-strand cDNA [[5,7,8], and [10]]. Thus, APOBEC3G is a member of a group of innate cellular antiviral response factors that limit the damage inflicted by viruses to their hosts (Figure 1).

**Figure 1:**
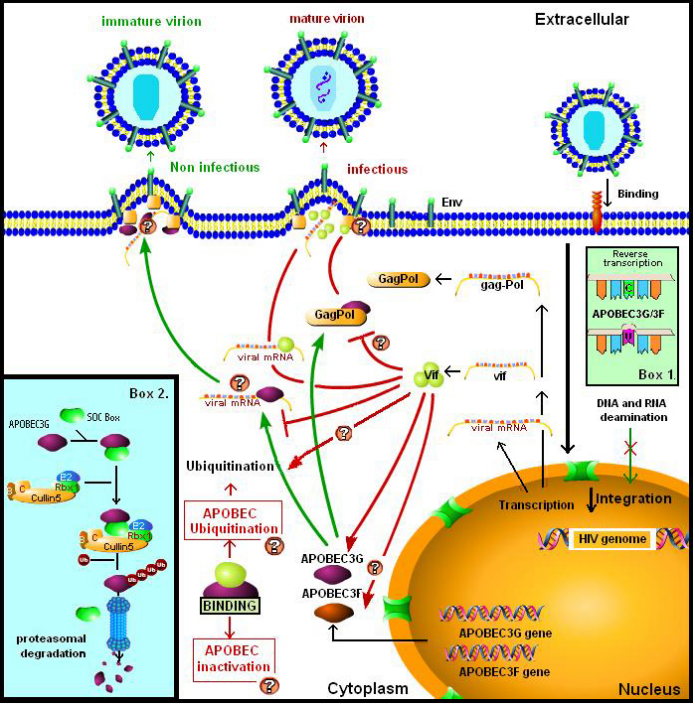
Schematic representation of Vif and APOBEC3G interactions during the HIV-1 replication cycle. Red arrows represent Vif action during the HIV-1 viral replication in non-permissive cells. Green arrows represent APOBEC3G/3F action in viral HIV-1 Vif defective virus. Broken arrows represent inhibition of APOBEC3G activity by Vif. Question marks (?) represent unresolved questions about Vif and APOBEC interactions. Box1: Schematic representation of minus-strand DNA and/or viral RNA deamination by APOBEC3G/3F [48]. Box2: Degradation model of APOBEC3G induced by Vif; Vif interacts with APOBEC3G as part of a Vif-Cul5-SCF complex resulting in the polyubiquitination and proteasomal degradation of APOBEC3G. Vif may have been derived from a cellular SOCS box protein that targets APOBEC 3G to the ECS ubiquitin ligase [49]. Two possible pathways of APOBEC3G regulation by Vif are represented.

The effects of APOBEC3G and its G-to-A deaminase activity on the survival of wild type HIV-1 *vif*+ virus are not known; but, current observations are that APOBEC3G confers a major deleterious effect to the HIV-1 genome when the Vif protein is absent. Historically, Vif has been known to play a dramatically important role in HIV-1 infectivity [12,13]. Vif is a basic protein of 23 kDa which is packaged into virions and which is required in virus producing cells during the late stages of infection to enhance viral infectivity by 10-to-1000 folds [14-17]. HIV-1 *vif*-defective virus can replicate in some permissive cells such as Jurkat and SupT1 cells, but cannot replicate in other non-permissive cells such as macrophages, primary human T cells, and some restrictive T cell lines [18-20]. For a very long time, it was not known what determined the difference between a permissive versus a non-permissive cell. The answer to this long-standing puzzle came when Michael Malim's laboratory found that non-permissive cells contain the anti-viral cellular factor APOBEC3G, and that the anti-viral action of APOBEC3G is thwarted by Vif [4].

Following on the heels of that initial observation, an enormous amount of effort emerged from several laboratories directed at elucidating how Vif mechanistically counteracts APOBEC3G in order to protect HIV-1 (Figure 1). Subsequent results showed remarkably that APOBEC3G binds Gag nucleocapsid NC protein, and in the absence of Vif, it is incorporated into the viral particle in close proximity to the reverse transcription complex [21]. Whether this interaction explains previous results on viral core stability or downstream effects during reverse transcription remains unclear [22]. Additionally, it was shown that Vif inhibited translation of APOBEC3G and/or its intracellular half-life [23-29]. In this regard, elegant biochemical studies showed that Vif interacted with APOBEC3G as part of a Vif-Cul5-SCF complex which led to the polyubiquitination and proteasomal degradation of APOBEC3G [30]. These latter results provided the mechanistic basis for the current accepted paradigm whereby increased degradation and/or reduced ambient level of APOBEC3G caused by Vif hinders the incorporation of APOBEC3G into virions. This consequently leads to an absence of APOBEC3G during reverse transcription in the virion-infected target cell, thereby permitting HIV-1 to replicate more robustly (Figure 1).

Now the report by *Kao et al*. adds a new wrinkle to this model by demonstrating that production of infectious human immunodeficiency virus type 1 does not require physical depletion of APOBEC3G in the presence of Vif from virus-producing cells [1]. Kao's study is remarkable for the fact that it raises the possibility of an alternative mechanism of viral protection from APOBEC3G by Vif. Indeed, some previous studies have shown drastic effects of Vif on steady-state amounts of APOBEC3G while others have found only modest effects [4,25-29]. Using confocal microscopy, Strebel and co-workers directly compared different methods of immunofluorescence to evaluate the expression of APOBEC3G at the single-cell level in absence or presence of Vif. Strikingly, depending on the fixation method and antibodies used, the results obtained showed variations in the number of cells which express APOBEC3G and Vif concomitantly. Thus, it is conceivable that direct binding of Vif to APOBEC3G may have alterred the deaminase's conformation, covering epitopes recognized by some of the antibodies used to detect APOBEC3G. Notably, most published studies have used APOBEC3G tagged at its N-terminus or C-terminus. Nevertheless, it should be kept in mind that a possible conformational change in APOBEC3G triggered by Vif-binding may also expose hydrophobic domains that are recognized by the ubiquitination and/or degradation machinery [31,32]. Thus, ubiquitination of APOBEC3G may still occur under the conditions used by *Kao et al*, but as demonstrated by these authors no degradation of APOBEC3G ensued. In this respect, protein ubiquitination could be not only a signal for protein turnover, but also a signal for cellular localization. An illustrative example of this concept is the putative ubiquitination of p6 protein in which findings by *Strack et al*. suggested that the engagement of the ubiquitin conjugation machinery by L domains plays a crucial role in the release of enveloped virus [33].

Many examples of allosteric alteration by protein-protein interaction are reported in the literature, and further work is necessary to evaluate possible conformational switches induced by Vif [34-36]. Bearing this in mind, it is noteworthy that a single amino acid substitution from D (aspartate)128 to K (lysine) in APOBEC3G can render this protein resistant to depletion by HIV-1 Vif [37-40]. It is possible that this amino acid represents a direct contact point for Vif, or that a change at this position influences the global conformation of the enzyme. Previous studies support the notion that this amino acid is positioned in a protein loop and is suitable for protein contact [38]. Thus, it is possible to speculate that Vif interaction with APOBEC3G at this position might alter protein conformation changing its biochemical and biophysical properties in ways that are larger than that normally expected from altering just one amino acid position in a protein. Elegant studies on the involvement of D128 in species specificity of Vif to counteract APOBEC3G function are, in part, consistent with this hypothesis [37-40]. Additionally, the D to K amino acid change at position 128 of APOBEC3G may alter the negative electrostatic interaction of aspartate to a positively charged amino acid which may inhibit Vif-induced allosteric changes. Conversely, if APOBEC3G from African green monkey, AGM, cells is considered, the positively charged lysine 128 found in this protein cannot interact with HIV-1 Vif but may instead do so with SIVAGM Vif, probably by using a negatively charged protein pocket. Thus, one idea is that conformational changes can result only from species-specific interaction between Vif and its cognate APOBEC3G. Although this model may be attractive, further refinements should be investigated since the isoelectric points of HIV-1 Vif and SIVAGM Vif are similar. Indeed, this assumption could be tested by studies similar to those of *Kao et al *using APOBEC3G with SIVAGM Vif or HIV-2 Vif, together with the role of these proteins in the context of APOBEC3F [41,42].

In the work of *Kao et al*., the authors explored the possibility that the different expression systems used by them and others could explain the discrepant results obtained on Vif-induced APOBEC3G depletion. For this hypothesis to hold several factors may be envisioned to interfere with the APOBEC3G-depletion mechanism of Vif. For example, one way to stabilize and activate p53 in cells is by interfering either with the interaction of MDM2 and p53 or with the ability of MDM2 to target its bound p53 for degradation [34,35]. Making a parallel between MDM2-p53 and Vif-APOBEC3G, two mechanisms can be hypothesized: one through changes in both proteins due to covalent modifications, and the other through non-covalent regulation of Vif-APOBEC3G association. In the case of MDM2-p53, it is apparent that both mechanisms are observed under different experimental conditions: induced phosphorylation of p53 can attenuate the p53-MDM2 interaction, and alternatively the human protein p14^ARF ^can bind to MDM2 and prevent its destruction of p53. Interestingly, these two mechanisms of p53 regulation appear to be entirely independent of each other, and emanated through distinct signal pathways. Using this parallel, one cautions that the findings of *Kao et al*. of a lack of APOBEC3G depletion do not rule out the possibility that Vif, under different conditions, can mediate proteasome dependent degradation of this deaminase [1].

Further research directions can be designed to evaluate additional putative regulatory mechanisms of APOBEC3G activity. For example, exposure of cells to a variety of extracellular stimuli leads to the rapid phosphorylation, ubiquitination, and ultimately proteolytic degradation of cellular proteins like IkappaB, which frees NF-kappaB to translocate to the nucleus where it regulates gene transcription [36]. NF-kappaB activation represents a paradigm for controlling the function of a regulatory protein via ubiquitination-dependent proteolysis, as an integral part of a phosphorylation based signaling cascade. After phosphorylation, the IKK phosphoacceptor sites on IkappaB serve as an essential part of a specific recognition site for E3RS (IkappaB/beta-TrCP), a SCF-type E3 ubiquitin ligase, thereby explaining how IKK controls IkappaB ubiquitination and degradation. A parallel may be envisaged for the regulation of Vif-induced APOBEC3G ubiquitination and the consequent depletion. It was reported recently that Vif is monoubiquitinated in the absence of APOBEC3G [28]. In addition, when Vif is co-expressed with APOBEC3G it is polyubiquitinated and rapidly degraded, suggesting that co-expression accelerates the degradation of both proteins [28,43]. Furthermore, mutations of conserved phosphorylation sites in Vif impair viral replication but do not affect APOBEC3G degradation, suggesting that Vif is important for other functions in addition to inducing proteasomal degradation of APOBEC3G. Whether or not phosphorylation regulates polyubiquitination or monoubiquitination is another open question.

*Kao et al*. interestingly also reported that expression of Vif from a codon-optimized vector had a more pronounced effect on APOBEC3G steady-state levels than wild-type Vif from pNL-A1 [1,44]. Even though the expression level of Vif-optimized construct is lower than wild-type, it is conceivable that its intracellular half-life may be increased affecting the quality and constancy of Vif-APOBEC3G association. Supporting this hypothesis are results where the authors showed a partial co-localization of Vif and APOBEC3G which may be indicative of higher k_off_, typically resulting from a lower protein affinity. This type of finding will not be observable by physical interaction assays which employ Western blotting since only stronger bindings are detected by such technique. *Kao et al*. remarkably demonstrated that infectious viruses are obtained in the presence of various ratios of APOBEC3G and Vif. Nevertheless, the question of whether under their conditions APOBEC3G and/or Vif are incorporated into viral particles remains pertinent. If the deaminase is not incorporated into the viral particle, then Vif may directly or indirectly inhibit the interaction of APOBEC3G with Gag polyproteins in the cytoplasm [45,46]. If APOBEC3G is included in the virion, then a direct blocking of its cytidine deaminase activity by Vif can be hypothesized. To date, a direct blocking of cytidine deaminase activity in the cytoplasm that consequently inhibits APOBEC3G interaction cannot be excluded. In fact, our own studies with a bacterial deaminase system where Vif and APOBEC3G are co-expressed show that Vif-mediated interaction with APOBEC3G can inhibit its cytidine deaminase activity (Santa-Marta *et al*; manuscript submitted). These results strongly support a new mechanistic function of HIV-1 Vif protein, complementing the model where Vif counteracts the inhibitory effects of APOBEC3G by enhancing its degradation via ubiquitin-proteasome pathway (Figure 1). The findings reported by *Kao et al*. together with the direct effects of Vif on the activity of cytidine deaminase (our work) may indicate an alternative protective mechanism used by HIV-1 to eliminate innate cellular immunity. Nevertheless, we cannot exclude that HIV-1 uses Vif to exert multiple mechanisms to synergistically and more effectively inhibit the anti-viral activity of APOBEC3G.

In conclusion, the existence of two different mechanisms may represent two faces of the same coin, with the common goal of inhibiting APOBEC3G's anti-viral activity. As is often the case with new findings, new questions are posed. The link between APOBEC3G's enzymatic function, its degradation pathway, and its incorporation into virions in the presence of Vif is certainly to require additional attention. Answers to these questions are likely to keep many of us busy for the foreseeable future.

## Competing Interests

None declared.

## Abbreviations

The abbreviations used are: HIV-1, human immunodeficiency virus, type 1; Vif, Viral Infectivity Factor; SIV, simian immunodeficiency virus; NC, nucleocapsid protein; APOBEC3G, apolipoprotein B mRNA-editing enzyme catalytic polypeptide-like 3G; APOBEC3F, apolipoprotein B mRNA-editing enzyme catalytic polypeptide-like 3F PBMC, peripheral blood mononuclear cells; Cul5, Cullin type 5; SCF, skp1-cullin-F-box protein ligase.
